# Surveillance for Coccidioidomycosis, Histoplasmosis, and Blastomycosis During the COVID-19 Pandemic — United States, 2019–2021

**DOI:** 10.15585/mmwr.mm7311a2

**Published:** 2024-03-21

**Authors:** Samantha L. Williams, Dallas J. Smith, Kaitlin Benedict, Jamie R. Ahlers, Connie Austin, Rachael Birn, Angel M. Carter, Natalie N. Christophe, Katie Cibulskas, Paul R. Cieslak, Suzanne N. Gibbons-Burgener, Michael Gosciminski, Malia J. Ireland, Katelyn V. Lazenby, Tom Loftus, Kristy Lunquest, Abby A. Mathewson, Alyssa D. Nguyen, Hanna N. Oltean, BreAnne Osborn, Erin M. Petro, Danny J. Power, Rebecca R. Reik, Levi Schlosser, Judi Sedivy, Chad B. Smelser, Tom Chiller, Mitsuru Toda

**Affiliations:** ^1^Mycotic Diseases Branch, Division of Foodborne, Waterborne, and Environmental Diseases, CDC; ^2^Delaware Department of Health and Social Services; ^3^Illinois Department of Public Health; ^4^Nebraska Department of Health and Human Services; ^5^Department of Environmental, Agricultural & Occupational Health, University of Nebraska Medical Center, Omaha, Nebraska; ^6^Kentucky Department for Public Health; ^7^Louisiana Department of Health; ^8^Ohio Department of Public Health; ^9^Oregon Health Authority; ^10^Division of Public Health, Wisconsin Department of Health Services; ^11^Rhode Island Department of Health; ^12^Minnesota Department of Health; ^13^Arkansas Department of Health; ^14^Indiana Department of Health; ^15^Maryland Department of Health; ^16^New Hampshire Department of Health and Human Services; ^17^California Department of Public Health; ^18^Washington State Department of Health; ^19^Utah Department of Health and Human Services; ^20^Kansas Department of Health and Environment; ^21^Montana Department of Public Health and Human Services; ^22^Michigan Department of Health and Human Services; ^23^North Dakota Health and Human Services; ^24^Pennsylvania Department of Health; ^25^New Mexico Department of Health.

**Summary**

**What is already known about this topic?**

Coccidioidomycosis, histoplasmosis, and blastomycosis, fungal diseases that can cause severe respiratory illness or disseminated disease and death, are underdiagnosed and underreported.

**What is added by this report?**

Coccidioidomycosis and histoplasmosis case counts declined in 2020 compared with 2019, then increased in 2021. These case fluctuations, a high 2021 blastomycosis case fatality rate (17%), and atypical 2020 seasonality across diseases suggest that these infections might have been affected by changes in health care–seeking behavior, diagnostic testing, or underreporting related to the COVID-19 pandemic.

**What are the implications for public health practice?**

Increased clinician education for coccidioidomycosis, histoplasmosis, and blastomycosis, and integration of diagnostic guidance and informational resources for fungal diseases into broader respiratory disease awareness and preparedness efforts, might improve timely diagnosis, patient management, and outcomes.

## Abstract

Coccidioidomycosis, histoplasmosis, and blastomycosis are lower respiratory tract fungal infections whose signs and symptoms can resemble those of other respiratory illnesses, including pneumonia caused by bacterial or viral etiologies; this overlap in clinical presentation might lead to missed or delayed diagnoses. The causative fungi live in the environment, often in soil or plant matter. To describe the epidemiologic characteristics of cases of coccidioidomycosis, histoplasmosis, and blastomycosis during the COVID-19 pandemic, CDC analyzed case surveillance data for 2019–2021. During this period, a total of 59,655 coccidioidomycosis cases, 3,595 histoplasmosis cases, and 719 blastomycosis cases were reported to CDC. In 2020, fewer cases of each disease occurred in spring compared with other seasons, and most cases occurred in fall; national seasonality is not typically observed, and cases were seasonally distributed more evenly in 2019 and 2021. Fewer cases coinciding with the start of the COVID-19 pandemic, along with an unusually high blastomycosis case fatality rate in 2021 (17% compared with more typical rates of 8%–10%), suggest that the pandemic might have affected patients’ health care–seeking behavior, public health reporting practices, or clinical management of these diseases. Increased awareness and education are needed to encourage health care providers to consider fungal diseases and to identify pneumonia of fungal etiology. Standardized diagnostic guidance and informational resources for fungal testing could be incorporated into broader respiratory disease awareness and preparedness efforts to improve early diagnosis of coccidioidomycosis, histoplasmosis, and blastomycosis.

## Introduction

Coccidioidomycosis, histoplasmosis, and blastomycosis are fungal infections that cause illness with primarily respiratory presentation ranging from mild symptoms to severe pulmonary or disseminated disease. The causative fungi live in soil and plant matter, and transmission usually occurs through inhalation of aerosolized spores. These diseases are typically limited to specific geographic ranges, but these ranges might be expanding because of climate change (*1*). Coccidioidomycosis primarily occurs in the southwestern United States, whereas histoplasmosis and blastomycosis are more commonly acquired in central and eastern states (*2*).

Diagnosis of these infections is challenging. Clinical signs and symptoms commonly resemble those of other respiratory infections, including COVID-19 and bacterial or viral community-acquired pneumonia, and laboratory tests might be difficult to access and interpret. Consequently, patients commonly can experience missed or delayed diagnosis and treatment, which can lead to persistent symptoms, severe disease, and adverse health outcomes (*3*).

Coccidioidomycosis, histoplasmosis, and blastomycosis are reportable in certain states (*4*). Each state designates reportable diseases, mandating health care providers and laboratories to notify public health departments of diagnosed cases or positive laboratory test results. Cases are classified according to the Council of State and Territorial Epidemiologists’ case definitions.* For nationally notifiable diseases such as coccidioidomycosis, states and the District of Columbia voluntarily submit case data to CDC through the National Notifiable Diseases Surveillance System (NNDSS).

The 2019 U.S. surveillance data for these fungal infections indicated that males and certain racial and ethnic populations were disproportionately affected. No pronounced national seasonality was observed. Histoplasmosis and blastomycosis were associated with high hospitalization and case fatality rates (*5*).

During the COVID-19 pandemic, many persons avoided or delayed seeking medical care because of concerns regarding disease transmission and busy medical facilities (*6*). Although few cases of coinfection with COVID-19 and coccidioidomycosis, histoplasmosis, or blastomycosis have been reported, how the COVID-19 pandemic might have otherwise affected acquisition, diagnosis, patient outcomes, and reporting of these diseases is not known (*7*,*8*). This report summarizes 2019–2021 U.S. surveillance data for coccidioidomycosis, histoplasmosis, and blastomycosis and examines epidemiologic changes that occurred during the COVID-19 pandemic compared with prepandemic data.

## Methods

### Data Sources

Case-level coccidioidomycosis data were submitted to NNDSS by 26 states and the District of Columbia.^†^ Aggregate case data, including hospitalizations and deaths, were submitted for histoplasmosis by 13 states^§^ and for blastomycosis by five states^¶^ (*4*). Death data were based on vital records; coccidioidomycosis-associated hospitalizations and deaths are not captured through NNDSS.

### Analysis

Confirmed coccidioidomycosis cases and confirmed and probable histoplasmosis and blastomycosis cases were included. Descriptive analyses of case counts, sex, age, race and ethnicity, and event month (the earliest known month associated with the illness, which could correspond to symptom onset, diagnosis, or laboratory testing) were performed. To better illustrate the potential impact of the COVID-19 pandemic, 2019 case counts, seasonality, hospitalizations, and deaths are presented in the results section to compare 2020–2021 cases with prepandemic data; demographic results include 2020–2021 data only to reflect the most up-to-date numbers. State-specific incidence (cases per 100,000 population) by sex, age, and race and ethnicity were calculated using 2020 and 2021 U.S. Census Bureau data. Univariate incidence rate ratios were calculated using robust Poisson models to compare different demographic groups with the referent population. Analyses were completed in RStudio (version 4.0.3; RStudio). This activity was reviewed by CDC, deemed not research, and was conducted consistent with applicable federal law and CDC policy.**

## Results

During 2019–2021, a total of 59,655 coccidioidomycosis cases (2019 = 20,061; 2020 = 19,284; 2021 = 20,320), 3,595 histoplasmosis cases (2019 = 1,124; 2020 = 1,012; 2021 = 1,459), and 719 blastomycosis cases (2019 = 240; 2020 = 238; 2021 = 241) were reported (Table 1). During 2020, fewer cases of each disease occurred in the spring than in other seasons (Supplementary Figure 1; https://stacks.cdc.gov/view/cdc/148381), whereas cases were seasonally distributed more evenly throughout 2019 and 2021 (Figure). In 2020, a higher percentage of total coccidioidomycosis and histoplasmosis cases occurred in winter and fall compared with other seasons, and the highest percentage of blastomycosis cases occurred during summer and fall.

**TABLE 1:** Number and percentage of coccidioidomycosis, histoplasmosis, and blastomycosis cases, by selected patient characteristics and clinical outcomes — United States, 2019–2021

Characteristic	No. (%)								
	Coccidioidomycosis*			Histoplasmosis^†^			Blastomycosis^§^		
	2019	2020	2021	2019	2020	2021	2019	2020	2021
**Sex**									
Female	9,638 (48)	9,108 (47)	9,330 (46)	488 (44)	410 (41)	606 (42)	71 (30)	87 (37)	86 (36)
Male	10,392 (52)	10,132 (53)	10,966 (54)	631 (56)	601 (59)	851 (58)	168 (70)	151 (63)	155 (64)
**Age group, yrs**									
<5	67 (<1)	77 (<1)	43 (<1)	6 (1)	13 (1)	9 (1)	2 (<1)	1 (<1)	0 (—)
5–19	1,252 (6)	1,056 (5)	981 (5)	102 (9)	116 (12)	168 (12)	22 (9)	25 (11)	17 (7)
20–39	4,695 (23)	4,466 (23)	4,346 (21)	287 (26)	233 (23)	384 (26)	75 (31)	56 (24)	49 (20)
40–64	8,184 (41)	7,832 (41)	8,321 (41)	482 (43)	423 (42)	578 (40)	93 (39)	106 (45)	109 (45)
65–79	4,520 (23)	4,573 (24)	5,055 (25)	202 (18)	203 (20)	274 (19)	42 (18)	43 (18)	58 (24)
≥80	1,323 (7)	1,256 (7)	1,533 (8)	35 (3)	24 (2)	41 (3)	6 (3)	7 (3)	8 (3)
**Race and ethnicity**									
AI/AN, NH	241 (3)	242 (3)	263 (2)	4 (<1)	<7 (1)	4 (<1)	10 (5)	10 (5)	8 (4)
Asian and NH/OPI, NH	450 (6)	346 (4)	578 (5)	25 (3)	15 (2)	22 (2)	15 (7)	19 (9)	7 (3)
Black or African American, NH	528 (7)	539 (7)	798 (7)	79 (9)	88 (11)	121 (10)	24 (12)	27 (12)	20 (9)
White, NH	3,252 (41)	3,870 (48)	5,141 (47)	656 (76)	6,01 (75)	9,08 (78)	144 (69)	142 (65)	163 (77)
Hispanic or Latino (all races)	2,559 (33)	2,665 (33)	3,424 (32)	63 (7)	55 (7)	68 (6)	14 (7)	21 (10)	14 (7)
Other^¶^	816 (10)	357 (4)	632 (6)	32 (4)	35 (4)	42 (4)	1 (<1)	1 (<1)	2 (<1)
Season**									
Winter	4,797 (24)	5,623 (29)	5,849 (29)	210 (24)	307 (31)	347 (24)	35 (22)	51 (22)	66 (28)
Spring	4,631 (23)	3,468 (18)	4,957 (24)	223 (25)	204 (20)	390 (27)	38 (23)	47 (20)	58 (24)
Summer	4,860 (24)	4,396 (23)	4,787 (24)	201 (23)	205 (20)	340 (24)	46 (29)	60 (25)	58 (24)
Fall	5,601 (28)	5,797 (30)	4,727 (23)	253 (29)	290 (29)	347 (24)	42 (26)	78 (33)	58 (24)
**Hospitalized**									
Yes	—	—	—	249 (54)	386 (46)	515 (46)	147 (65)	138 (66)	143 (63)
No	—	—	—	211 (46)	451 (54)	602 (54)	81 (35)	70 (34)	85 (37)
**Outcome**									
Died	—	—	—	20 (5)	37 (6)	51 (6)	20 (9)	20 (9)	40 (17)
Survived	—	—	—	395 (95)	621 (94)	799 (94)	204 (91)	191 (91)	190 (83)
**Total**	**20,061**	**19,284**	**20,320**	**1,124**	**1,012**	**1,459**	**240**	**238**	**241**

**Abbreviations:** AI/AN = American Indian or Alaska Native; NH = non-Hispanic; NH/OPI = Native Hawaiian or other Pacific Islander.

* Sex was missing for 95 cases, age was missing for 85 cases, and race and ethnicity were missing for 32,964 cases.

^†^ Sex was missing for 10 cases, age was missing for 15 cases, race and ethnicity were missing for 762 cases, season was missing for 278 cases, hospitalization was missing for 1,181 cases, and death was missing for 1,672 cases.

^§^ Sex was missing for one case, race and ethnicity were missing for 80 cases, season was missing for 82 cases, hospitalization was missing for 55 cases, and death was missing for 54 cases.

^¶^ Calculated on basis of two or more reported race categories.

** Seasons defined as winter (December–February), spring (March–May), summer (June–August), and fall (September–November).

**FIGURE:**
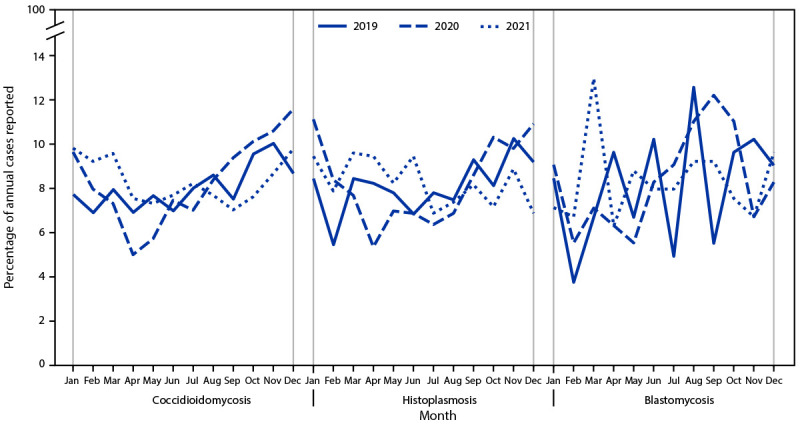
Percentage of reported annual coccidioidomycosis, histoplasmosis, and blastomycosis cases,* by month of report — United States, 2019–2021 * Denominator is the total number of cases reported in each year (2019, 2020, and 2021) with a known earliest recorded event month for each fungal disease.

During 2020–2021, the majority of cases of all three diseases occurred in males (coccidioidomycosis = 53%; histoplasmosis = 59%; blastomycosis = 64%), and the highest percentages occurred among persons aged 40–64 years (coccidioidomycosis = 16,153 [41%]; histoplasmosis = 1,001 [41%]; blastomycosis = 215 [45%]) (Table 1); incidences of all diseases were slightly higher among persons aged 65–79 years (coccidioidomycosis = 27.3 per 100,000; histoplasmosis = 2.5; blastomycosis = 1.3) (Table 2).

**TABLE 2:** Incidence* of coccidioidomycosis, histoplasmosis, and blastomycosis, by selected patient characteristics — United States, 2019–2021

Characteristic	Incidence* (95% CI)								
	Coccidioidomycosis^†^			Histoplasmosis^§^			Blastomycosis^¶^		
	2019	2020	2021	2019	2020	2021	2019	2020	2021
**Sex**									
Female	14.1 (14.1–14.7)	12.4 (12.2–12.7)	12.6 (12.4–12.9)	1.3 (1.2–1.4)	1.1 (1.0–1.2)	1.6 (1.5–1.8)	0.5 (0.4–0.6)	0.3 (0.2–0.4)	0.6 (0.5–0.7)
Male	15.8 (15.5–16.2)	14.1 (13.8–14.4)	15.0 (14.7–15.3)	1.8 (1.6–1.9)	1.7 (1.6–1.8)	2.3 (2.2–2.5)	1.2 (1.0–1.4)	1.3 (1.2–1.5)	1.1 (0.9–1.2)
**Age group, yrs**									
<5	0.8 (0.7–1.1)	0.9 (0.7–1.2)	0.5 (0.4–0.7)	0.1 (0.1–0.3)	0.3 (0.2–0.5)	0.2 (0.1–0.4)	0.1 (0–0.5)	0.1 (0–0.4)	0 (—)
5–19	4.9 (4.7–5.2)	4.0 (3.7–4.2)	3.6 (3.4–3.9)	0.7 (0.6–0.9)	0.8 (0.7–1.0)	1.2 (1.0–1.4)	0.4 (0.3–0.6)	0.5 (0.3–0.7)	0.3 (0.2–0.5)
20–39	12.9 (12.6–13.3)	11.9 (11.5–12.2)	11.6 (11.2–11.9)	1.5 (1.3–1.7)	1.2 (1.1–1.4)	2.0 (1.8–2.2)	1.0 (0.8–1.2)	0.7 (0.6–1.0)	0.6 (0.5–0.9)
40–64	19.8 (19.3–20.2)	18.0 (17.6–18.4)	18.9 (18.5–19.4)	2.0 (1.9–2.2)	1.8 (1.7–2.0)	2.5 (2.3–2.7)	1.0 (0.8–1.2)	1.1 (0.9–1.4)	1.2 (1.0–1.4)
65–79	27.3 (26.6–28.1)	27.5 (26.7–28.3)	27.6 (26.8–28.4)	2.1 (1.9–2.5)	2.2 (2.0–2.6)	2.7 (2.4–3.1)	1.1 (0.8–1.5)	1.2 (0.9–1.6)	1.4 (1.1–1.9)
≥80	26.2 (24.8–27.7)	23.8 (22.5–25.1)	30.0 (28.6–31.6)	1.1 (0.8–1.6)	0.8 (0.5–1.2)	1.5 (1.1–2.0)	0.5 (0.2–1.1)	0.6 (0.3–1.3)	0.7 (0.4–1.4)
**Race and ethnicity**									
AI/AN, NH	17.4 (15.3–19.7)	18.4 (16.2–20.8)	25.6 (22.7–28.9)	1.2 (0.5–3.2)	2.4 (1.1–5.0)	2.0 (0.8–5.4)	4.5 (2.4–8.4)	5.0 (2.7–9.3)	6.1 (3.0–12.1)
Asian and NH/OPI, NH	4.6 (4.2–5.0)	3.4 (3.1–3.8)	5.8 (5.4–6.3)	1.0 (0.7–1.5)	0.6 (0.3–0.9)	0.9 (0.6–1.3)	1.6 (1.1–2.4)	2.0 (1.3–3.1)	0.8 (0.4–1.6)
Black or African American, NH	4.0 (3.7–4.4)	3.9 (3.6–4.3)	5.9 (5.5–6.4)	0.9 (0.7–1.1)	1.0 (0.8–1.2)	1.4 (1.2–1.7)	0.6 (0.4–1.0)	0.7 (0.5–1.0)	0.5 (0.3–0.8)
White, NH	4.1 (4.0–4.2)	4.4 (4.3–4.6)	5.9 (5.7–6.0)	1.3 (1.2–1.4)	1.2 (1.1–1.3)	1.7 (1.6–1.9)	0.7 (0.4–1.2)	0.7 (0.6–0.8)	0.8 (0.7–0.9)
Hispanic or Latino (all races)	11.2 (10.8–11.6)	9.8 (9.5–10.2)	12.5 (12.1–12.9)	1.2 (0.9–1.5)	0.8 (0.6–1.0)	1.0 (0.8–1.3)	0.9 (0.8–1.1)	1.1 (0.7–1.7)	0.8 (0.5–1.3)
Other**	4.6 (4.0–5.3)	5.2 (4.7–5.8)	8.6 (8.0–9.3)	1.9 (1.3–2.6)	1.1 (0.8–1.6)	1.3 (0.9–1.7)	0.2 (0–1.4)	0.1 (0–0.6)	0.1 (0–0.6)
**Total**	**15.1** **(14.9–15.3)**	**13.1** **(12.9–13.2)**	**13.8** **(13.6–14.0)**	**1.6** **(1.5–1.7)**	**1.4** **(1.3–1.5)**	**2.0** **(1.9–2.1)**	**0.8** **(0.7–0.9)**	**0.8** **(0.7–0.9)**	**0.8** **(0.7–0.9)**

**Abbreviations:** AI/AN = American Indian or Alaska Native; NH = non-Hispanic; NH/OPI = Native Hawaiian or other Pacific Islander.

* Cases per 100,000 population. Summed state-specific denominators from 2019–2021 U.S. Census Bureau data were used to calculate incidence for the total grouping for each year.

^†^ Sex was missing for 68 cases, age was missing for 65 cases, and race and ethnicity were missing for 20,749 cases.

^§^ Sex was missing for five cases, age was missing for five cases, and race and ethnicity were missing for 497 cases.

^¶^ Race and ethnicity were missing for 48 cases.

** Calculated on the basis of two or more reported race categories.

Data on race and ethnicity during 2020–2021 were available for 48% of coccidioidomycosis, 80% of histoplasmosis, and 90% of blastomycosis cases. Compared with incidence in non-Hispanic White (White) persons (0.7 per 100,000), blastomycosis incidence was approximately eight times higher among non-Hispanic American Indian or Alaska Native (AI/AN) persons (5.5) and twice as high among non-Hispanic Asian and Native Hawaiian or other Pacific Islander (A/NHOPI) populations (1.4). Compared with incidence among White persons (5.1) coccidioidomycosis incidence was more than four times as high among AI/AN persons (22.0) and twice as high among Hispanic or Latino (Hispanic) persons (11.1). Histoplasmosis incidence among AI/AN persons (2.2) was 1.6 times as high as that among White persons (1.4) (Supplementary Figure 2; https://stacks.cdc.gov/view/cdc/148382) (Supplementary Table; https://stacks.cdc.gov/view/cdc/148380).

Among cases for which hospitalization data were available during 2019–2021 (67% of histoplasmosis and 92% of blastomycosis cases), 1,150 (48%) patients with histoplasmosis and 428 (64%) with blastomycosis were hospitalized. Among those with available mortality data (53% of histoplasmosis cases and 92% of blastomycosis cases), 108 (6%) histoplasmosis patients and 80 (14%) blastomycosis patients died (Table 1). Although the histoplasmosis case fatality rate (CFR) remained stable from 2020 to 2021, the blastomycosis CFR nearly doubled from 9% in 2019 and 2020 to 17% in 2021.

## Discussion

Coccidioidomycosis, histoplasmosis, and blastomycosis caused substantial illness nationwide during 2019–2021. The predominance among males, older adults, and AI/AN persons aligns with previous data and historical trends (*5*). Yearly case count fluctuations during 2019–2021, changes in seasonality, and increase in the blastomycosis CFR in 2021 were atypical and are potentially related to the COVID-19 pandemic, which might have affected acquisition, diagnosis, management, and reporting of these three fungal diseases. Seasonality is not typically observed nationally for these diseases, and the low percentage of cases observed during spring 2020 compared with spring 2019 and spring 2021 might be related to reduced health care–seeking behavior associated with concerns about potential COVID-19 transmission in health care settings. This delay or avoidance of medical care, which was prevalent in the early months of the pandemic (*6*), likely exacerbated misdiagnosis and diagnostic delays. Fewer reported coccidioidomycosis and histoplasmosis cases in 2020 compared with both 2019 and 2021 might also reflect changes in health care–seeking behavior, preventative measures for respiratory diseases such as mask-wearing, reduced travel to areas endemic for these fungal diseases, lower clinical suspicion of fungal infections given the focus on COVID-19, or underreporting by overwhelmed public health agencies (*5*). Until 2020, coccidioidomycosis cases had been consistently increasing each year since 2014.^††^ Compared with 2019, histoplasmosis case counts declined by 10% in 2020, but subsequently increased 44% in 2021 compared with 2020 (*5*). COVID-19 transmission concerns that prompted persons to spend more time outdoors in 2020 and 2021 than in 2019^§§^ might have increased exposure to pathogenic fungi. Further research is needed to understand the marked rise in reported histoplasmosis cases from 2020 to 2021.

Although in-hospital blastomycosis mortality has increased in recent years (*9*), the 2021 blastomycosis CFR (17%) was unusually high, particularly given the stable hospitalization rates during the reporting period, which was consistent with prepandemic rates; blastomycosis CFR generally ranges from 8%–10% (*5*,*9*). Diagnosis of blastomycosis is challenging because symptoms are nonspecific and the availability of laboratory tests is limited; diagnostic delays exacerbated by the pandemic might have impeded prompt management of blastomycosis or associated comorbidities, which could have led to more severe or disseminated disease during the pandemic. Some health departments noted that COVID-19 prompted better access to death reports and additional scrutiny of contributing causes of death, which might have influenced the observed CFR (*10*).

The racial and ethnic disparities observed in 2020–2021 generally align with those reported in 2019, with higher incidence of all three diseases among AI/AN persons as well as higher incidence of coccidioidomycosis among Hispanic persons and higher incidence of blastomycosis among A/NHOPI persons compared with incidence in White populations (*5*). Similar to coccidioidomycosis and blastomycosis, histoplasmosis incidence was also higher among AI/AN than White persons, which differed from 2019, when incidence was similar across racial and ethnic groups (*5*). How these racial and ethnic disparities might be affected by geographic, biologic, or sociodemographic differences is not clear. More complete and detailed race and ethnicity data are needed to better understand how these factors influence disease and to guide actionable public health responses.

### Limitations

The findings in this report are subject to at least three limitations. First, because of longstanding diagnostic challenges, case counts underestimate the actual number of coccidioidomycosis, histoplasmosis, and blastomycosis cases, and data are limited to the subset of states where each disease is reportable (*4*). Second, data related to potential exposures, underlying conditions, laboratory testing, clinical course, and treatment were not available, hindering the ability to distinguish the potential effects of the COVID-19 pandemic from other influences, including weather patterns, awareness, or testing and reporting practices. Data were incomplete for race and ethnicity, event month, hospitalization, and death, and the lack of event date standardization could lead to misclassification of monthly case counts. Finally, only aggregate-level data were available for histoplasmosis and blastomycosis, which precluded bivariate analyses.

### Implications for Public Health Practice

Increased awareness is needed to improve prompt diagnosis and treatment of coccidioidomycosis, histoplasmosis, and blastomycosis, particularly during periods of increased incidence of other respiratory diseases. To reduce misdiagnosis of these three fungal infections, standardized diagnostic guidance and informational resources for pan-respiratory testing, including fungal diseases, are needed and could be incorporated into broader respiratory disease awareness and preparedness efforts. Education to help clinicians distinguish fungal pneumonia from other respiratory infections might improve accurate diagnosis. Enhanced and expanded surveillance can also improve understanding of risk factors and epidemiologic trends to help guide efforts to raise awareness and improve diagnosis, management, and patient outcomes.

## Footnotes

* https://ndc.services.cdc.gov/case-definitions/coccidioidomycosis-2011/; https://ndc.services.cdc.gov/case-definitions/histoplasmosis-2017/; https://ndc.services.cdc.gov/case-definitions/blastomycosis-2020/

† Coccidioidomycosis cases reported for Alabama, Arizona, Arkansas, California, Delaware, District of Columbia, Indiana, Kansas, Louisiana, Maryland, Michigan, Minnesota, Missouri, Montana, Nebraska, Nevada, New Hampshire, New Mexico, North Dakota, Ohio, Oregon, Rhode Island, South Dakota, Utah, Washington, Wisconsin, and Wyoming.

§ Histoplasmosis cases reported for Arkansas, Delaware, Illinois, Indiana, Kansas, Kentucky, Louisiana, Michigan, Minnesota, Nebraska, Pennsylvania, Rhode Island, and Wisconsin.

¶ Blastomycosis cases reported for Arkansas, Louisiana, Michigan, Minnesota, and Wisconsin.

** 45 C.F.R. part 46.102(l)(2), 21 C.F.R. part 56; 42 U.S.C. Sect. 241(d); 5 U.S.C. Sect. 552a; 44 U.S.C. Sect. 3501 et seq.

††
https://www.cdc.gov/fungal/diseases/coccidioidomycosis/statistics.html

§§
https://outdoorindustry.org/wp-content/uploads/2015/03/2021-Outdoor-Participation-Trends-Report.pdf
